# Genetics of Anthracycline-Associated Cardiotoxicity

**DOI:** 10.3389/fcvm.2022.867873

**Published:** 2022-04-21

**Authors:** Talal Khalid Al-Otaibi, Benjamin Weitzman, Usman A. Tahir, Aarti Asnani

**Affiliations:** CardioVascular Institute, Beth Israel Deaconess Medical Center, Boston, MA, United States

**Keywords:** anthracycline associated cardiotoxicity, genetics, genetic testing, cardio-oncology, anthracycline

## Abstract

Anthracyclines are a major component of chemotherapies used in many pediatric and adult malignancies. Anthracycline-associated cardiotoxicity (ACT) is a dose-dependent adverse effect that has substantial impact on morbidity and mortality. Therefore, the identification of genetic variants associated with increased risk of ACT has the potential for significant clinical impact to improve patient care. The goal of this review is to summarize the current evidence supporting genetic variants associated with ACT, identify gaps and limitations in current knowledge, and propose future directions for incorporating genetics into clinical practice for patients treated with anthracyclines. We will discuss mechanisms of ACT that could be illuminated by genetics and discuss clinical applications for the cardiologist/cardio-oncologist.

## Introduction

Anthracyclines are among the most frequently used agents for treating cancer. They serve as the cornerstone for chemotherapy regimens commonly used to treat hematological and solid organ malignancies. Despite their efficacy, their utility is limited by a dose-dependent cardiomyopathy, also known as anthracycline-associated cardiotoxicity (ACT). ACT is commonly detected by imaging studies and may present as anthracycline-associated heart failure. It is estimated that one in ten children exposed to cumulative anthracycline doses >300 mg/m^2^ develop anthracycline-induced heart failure (1), and childhood cancer survivors are at a 5–10-fold increased risk of cardiac dysfunction compared to the general population (1, 2). Despite established risk factors such as older age and pre-existing cardiovascular comorbidities, there remains significant inter-individual variability in the development of cardiac toxicity. Some patients tolerate high doses without adverse effects, whereas others develop ACT at relatively low doses. The variable predisposition to ACT between patients suggests that genetic susceptibility may play a role in the development of ACT. Candidate genes with potential contributions to ACT pathophysiology have traditionally been nominated based on mechanistic studies in preclinical models, such as zebrafish and mice. While these models can provide valuable mechanistic data in the setting of *in vivo* treatment, conservation of the pathway and/or specific genetic variant of interest across species remains an important consideration in translating observations to human patients. Cell lines derived directly from human tissues, such as patient-derived human induced pluripotent stem cell-derived cardiomyocytes (hiPSC-CMs), offer a unique opportunity to identify genetic variation that may contribute to ACT risk in specific patients. Both animal models and hiPSC-CMs provide mechanistic information that is often complementary to discovery – omics studies in patients.

This review will address the current knowledge of specific genetic associations with ACT, current practice of genetic testing and interpretation in the cardiology clinic and the potential management implications of genetic testing, particularly related to overlap between ACT and other cardiomyopathies.

## Genomic Variants Associated With Anthracycline Associated Cardiotoxicity

Efforts to identify genetic variants associated with ACT have been conducted in several studies in pediatric and adult cohorts. Candidate gene approaches and genome wide association studies (GWAS) have yielded several single nucleotide polymorphisms (SNPs) of potential interest. In general, genetic studies in this patient population have been limited by their small sample size and the lack of consensus for clinical or echocardiographic diagnosis of ACT.

Surveillance protocols to detect ACT, as well as the definition of ACT, have not been standardized across studies. As a result, the incidence and timeline of diagnosis of ACT varies considerably in the literature. Clinically, ACT has been identified as new onset heart failure/cardiomyopathy in patients who were treated with anthracyclines. Left ventricular ejection fraction (LVEF) is commonly used in the detection of ACT, but echocardiography surveillance protocols vary considerably from one center to another, and asymptomatic cardiomyopathy is likely underdiagnosed. In addition, LVEF is influenced by preload, afterload and adrenergic state, leading to subjectivity and interpretive variation. A consensus definition of ACT lead by the American Society of Echocardiography (ASE) and the European Association of Cardiovascular Imaging (EACVI) defines ACT as a decrease in the LVEF >10% to an absolute value of <53% (3).

## Genes Associated With Anthracycline-Associated Cardiotoxicity According to Presumed Pathophysiologic Mechanism

Several genetic modifiers have been identified as potentially contributing to ACT, which we will review here according to their proposed pathophysiologic mechanism (summarized in Table 1).

**TABLE 1:** Summary of studies investigating the role of genetic modifiers implicated in anthracycline-associated cardiotoxicity.

Genes with SNP	SNP effect	Replication	Authors	Year	Cohort	Case	Control	Study population	Definition of cardiotoxicity	Anthracycline used
**DNA damage**										
RARG rs2229774	Predisposing	Replicated in similar cohorts	Aminkeng et al. (4)	2015	280 96 80	32 22 19	248 74 61	**Pediatric** ALL, AML, HL, NHL, osteosarcoma, rhabdomyosarcoma, Ewing sarcoma, hepatoblastoma, neuroblastoma, Wilms tumor	(i) LVEF < 45% (ii) Dilation of LV-end-diastolic dimension > 117%.	Doxorubicin, daunorubicin, epirubicin
**Anthracycline transportation and metabolism**										
SLC28A3 rs78537585	Protective	Replicated in two different cohorts	Visscher et al. (5)	2012	156 188 96	38 40 43	118 148 53	**Pediatric** ALL, AML, other leukemia, HL, NHL osteosarcoma, rhabdomyosarcoma, Ewing sarcoma, other sarcoma, Wilms tumor, hepatoblastoma, neuroblastoma, carcinoma	1. FS < 26% 2. Signs and symptoms indicating need for cardiac compromise intervention based on CTCAEv3	Doxorubicin, daunorubicin
UGT1A6 rs17863783	Predisposing	Replicated in same analysis	Visscher et al. (6)	2013	177	46	131	**Pediatric** ALL, AML, HL, NHL osteosarcoma, rhabdomyosarcoma, Ewing sarcoma, other sarcoma, Wilms tumor, hepatoblastoma, neuroblastoma, carcinoma, germ cell tumor	1. FS < 26% 2. Signs/symptoms of cardiac compromise indicating need for intervention based on CTCAEv3	Doxorubicin, daunorubicin
SULT2B1 rs10426377	Predisposing									
CBR3 rs1056892	Predisposing	No replication performed	Blanco et al. (7)	2012	487	170	317	**Pediatric** HL, NHL, bone tumors, soft tissue sarcoma, ALL, AML, other	1. Signs/symptoms of cardiac compromise based on AHA criteria 2. Echo evidence of LV dysfunction (LVEF < 40%; FS < 28%)	Not specified
ABCC1 rs3743527, rs246221, rs3743527	Predisposing	No replication performed	Semsei et al. (8)	2012	234	–	–	**Pediatric** ALL	Change in LV FS	Daunorubicin, doxorubicin/not reported
ABCC2 rs8187710	Predisposing	No replication performed	Armenian et al. (9)	2013	255	77	178	**Pediatric and Adult** Leukemia, myeloma, lymphoma status post-hematopoietic cell transplantation	Sign/symptoms of cardiac compromise indicating need for intervention based on AHA criteria	Not specified
**Antioxidant mechanisms:**										
HAS3 rs2232228	Predisposing	Replicated in an independent set of 76 patients	Wang et al. (10)	2014	287	93	194	**Pediatric and Adult** HL, NHL bone tumors, soft tissue sarcoma, ALL, AML, other	AHA criteria for cardiac compromise: 1. Symptoms/signs of cardiac compromise 2. Echo evidence of LV dysfunction (LVEF # 40% and/or FS # 28%)	Not specified
GSTM1 null genotype	Predisposing	No replication performed	Singh et al. (11)	2020		75	92	**Pediatric** ALL, AML, HL, NHL, bone tumors, kidney tumor, sarcoma, neuroblastoma	LVEF < 40% and/or FS < 28%	Not specified
NOS3 rs1799983	Protective	No replication	Krajinovic et al. (12)	2016	251	–	–	**Pediatric** ALL	Reduction in FS and EF	Doxorubicin
ABCC5 rs7627754	Predisposing	Replicated in 44 ALL patients								
RAC2 rs1305833	Predisposing	No replication performed	Armenian et al. (9)	2013	255	77	178	**Pediatric and Adult** Leukemia, myeloma, lymphoma status post-hematopoietic cell transplantation	Sign/symptoms of cardiac compromise indicating need for intervention based on AHA criteria	Not specified
**Sarcomere dysfunction**										
CELF4 rs1786814	Predisposing	Replicated in an independent set of patients	Wang et al. (13)	2016	331 54	112 54	219 0	**Pediatric** HL, NHL, sarcoma, AML, ALL, and others replication HL, NHL, sarcoma, AML, ALL, and others	1. Signs/symptoms of cardiac compromise based on AHA criteria 2. Absence of symptoms/signs with echo evidence of LV dysfunction (EF # 40% and/or FS # 28%)	Not reported
TTNtv	Predisposing	Preclinical replication performed	Garcia-Pavia et al. (14)	2019	213	–	–	**Adult** Breast cancer, AML, other solid tumor	Reduction in EF	Not reported

### DNA Damage

DNA topoisomerase I (Top1) and II (Top2) relieve tension in overwound DNA by introducing a single or double-stand DNA break. Anthracyclines target the Top2-cleaved DNA complex, causing accumulation of double-strand DNA breaks (15) ultimately leading to apoptosis. Cardiomyocyte-specific deletion of Top2b protected mice from the development of doxorubicin-induced progressive heart failure (16). Furthermore, disruption of Top2-beta using clustered regularly interspaced short palindromic repeats and associated protein 9 (CRISPR/Cas9) significantly reduced the sensitivity of hiPSC-CMs to doxorubicin-induced double stranded DNA breaks and cell death (17).

#### Retinoic Acid Receptor Gamma

Although specific genetic variants in Top2-beta have not been identified as associated with anthracycline cardiotoxicity in patients, supporting findings have emerged related to retinoic acid receptor gamma (RARG), which binds to the Top2-beta promoter and participates in DNA damage-associated cell death. RARG binds to DNA regulatory sequences called retinoic acid receptor elements (RAREs) and has been implicated in the development of anthracycline cardiomyopathy in a mouse model (16). Aminkeng et al. performed a GWAS in 280 pediatric patients treated for childhood cancer and identified SNP rs2229774 in RARG to be associated with ACT [odds ratio (OR) 4.7, *P* = 5.9 × 10^–8^] (4).

### Anthracycline Transportation and Metabolism

#### Solute Carrier

The solute carrier (SLC) super family of membrane proteins have been described to function as drug transporters for anthracyclines (18–20). Visscher et al. (6) observed consistent association of two variants of the SLC transport protein SLC28A3 (rs7853758 and rs885004) with resistance to ACT in several pediatric cohorts (*P* = 1.8 × 10^–5^; OR: 0.35). The association between rs7853758 and ACT was also observed in the Dutch-EKZ cohort (5, 21). The hypothesis is that the proteins encoded by these genes could transport anthracyclines into the cell leading to increased toxicity, whereas reduced function will be protective. The SLC22A17 variants rs4982753 and rs4149178 were also identified to be associated with ACT (6).

#### UGT1A6

Variants in UDP-glucuronosyltransferase family 1A6 (rs17863783, V209 V) have been associated with ACT in pediatric cohorts (5, 6). UGT1A6 plays a role in drug detoxification through the glucuronidation pathway (22). Although doxorubicin and daunorubicin are not themselves glucuronidated, certain downstream metabolites undergo glucuronidation (23). It can therefore by hypothesized that altered UGT1A6-mediated glucuronidation of anthracycline metabolites might lead to accumulation of toxic anthracycline metabolites in patients carrying UGT1A6*4, resulting in an increased risk of ACT.

#### Carbonyl Reductase 3

The alcohol metabolite of doxorubicin, doxorubicinol, is thought to be the primary mediator of the cardiotoxic effects of this agent. Carbonyl reductase (CBR) converts doxorubicin to doxorubicinol, leading to its accumulation in cardiomyocytes and a subsequent increase in cellular injury and death (7, 24, 25). These metabolites form a reservoir in cardiomyocytes and impair contractility through inhibition of Ca^2+^ and Na^+^/K^+^ pump activity (26). Myocardial accumulation of these metabolites has been associated with subsequent cardiomyopathy (27). CBRs catalyze cardiotoxic alcohol metabolites and are considered to be major anthracycline metabolizing enzymes. The role of CBRs were investigated by Blanco et al. (7) in pediatric cancer survivors. They found that individuals with CBR3 V244M homozygous G genotype (CBR3:GG) had an increased risk for cardiomyopathy associated with low to moderate dose anthracyclines (1–250 mg/m^2^). This is thought to be due to upregulated CBR3 expression mediated by nuclear transcription factor Nrf2, leading to increased synthesis of cardiotoxic anthracycline alcohol metabolites. However, involvement of these enzymes has not been as clearly established in other studies of ACT (4, 5, 9).

#### ABCC1, ABCC2, and ABCC5

ATP binding cassette (ABC) proteins are membrane-bound transporters involved in the clearance of anthracycline from the cell using energy derived from ATP hydrolysis (28). ABCC1 is highly expressed in the human heart. In mice, its expression is upregulated in the heart following exposure to doxorubicin (29). In a pediatric cohort with acute lymphoblastic leukemia, patients with SNP rs3743527 TT in ABCC1 had decreased LV fractional shortening at follow-up (8). The combination of either the TT or TC genotype was associated with decreased LV fractional shortening as well. Armenian et al. identified ABCC2 SNP rs8187710 to be associated with a 4.3-fold risk of ACT in a retrospective study of a mixed adult and pediatric cohort of patient receiving anthracycline prior to hematopoietic cell transplant (9). ABCC5 has been implicated in ACT with a TT genotype at SNP rs7627754 associated with reduced ejection fraction and fractional shorting in pediatric patients with ALL (12).

#### Sulfotransferase Family Cytosolic Member 2B1

Sulfotransferase family cytosolic member 2B1 (SULT2B1) is an enzyme that increases drug solubility in water and promotes renal excretion by conjugation of the sulfate group. A possible ACT association (*P* = 0.054) of the rs10426377 variant in SULT2B1 was reported by Visscher et al. (6). Interestingly, this sensitizing effect was noticed in men but not in women (6). It is possible that the SULT2B1 variant affects anthracycline catabolism and subsequent renal excretion, although additional data will be needed to determine the clinical significance of SULT2B1.

### Antioxidant Mechanisms

Oxidative stress has also been implicated in the mechanism of ACT. Cardiomyocytes are particularly vulnerable to ROS induced cellular damage. Anthracyclines are thought to form ROS through dysfunction of the mitochondrial electron transport chain and iron accumulation (30) and by dysregulation of cardiomyocyte autophagy (31).

#### Hyaluronan Synthase 3

Hyaluronan synthase 3 (HAS3) enzymes synthesize hyaluronan, a glycosaminoglycan which is found in the extracellular matrix and has a role in tissue remodeling post injury. In addition to hyaluronan’s tissue remodeling properties, it may also decrease reactive oxygen species–induced cardiac injury. Wang et al demonstrated in a pediatric cohort of cancer survivors that patients with the AA genotype in SNP rs2232228 of HAS3 who were exposed to high doses of anthracyclines (>250 mg/m^2^) were at 8.9-fold greater risk of developing ACT compared with those with the GG genotype (10).

#### GSTM1

Glutathione S-transferases (GST) represent a class of phase II detoxification enzymes that catalyze reduced glutathione and eventually lead to its elimination from the body (32). GST are also scavengers of free radicals, preventing oxidative damage. GSTM1 expression varies by race and ethnicity, with the GSTM1 null genotype identified more frequently in east Asians (70–79%) and less frequently in Europeans (5%) (33–35). In pediatric cancer survivors, Miranda et al. demonstrated an association between the GSTM1 null genotype and the risk of developing cardiomyopathy (11). Similarly, GSTP1 has been reported to be associated with ACT in two small studies (36, 37).

#### Nitric Oxide Synthase 3

Anthracyclines can bind to nitric oxide synthase 3 (NOS3) leading to the inhibition of its activity. In a mouse model, NOS3^–/–^ mice demonstrated less cardiotoxicity following doxorubicin exposure, whereas mice overexpressing wild type NOS3 exhibited more cardiotoxicity (38). Krajinovic et al. identified a NOS3 variant rs1799983 to be cardioprotective in a cohort of pediatric ALL patient exposed to doxorubicin (12).

#### NADPH Multienzymes Complex

Anthracyclines are lipophilic molecules which diffuse passively across cell membranes and into the mitochondria. During anthracycline reduction, a superoxide anion is formed. Polymorphism in NADPH oxidase subunits have been associated with the production of ROS. A SNP (rs13058338) in RAC2, which encodes a Rho-GTPase that regulates NADPH oxidase, has been associated with susceptibility to ACT (OR = 2.8, *P* < 0.01) (9). In a report by Wojnowski et al., NADPH oxidase (NOX2) knockout mice were protected against anthracycline induced heart failure (39).

### Alternative Splicing of TNNT2/Sarcomere Dysfunction

#### CELF4

The cytosine-uridine-guanine repeat binding protein (CUGBP) family are splicing regulators that control developmentally regulated tissue-specific splicing events. CELF4 is an RNA protein involved in pre-mRNA splicing, known to mediate the splicing of the gene TNNT2 that encodes cardiac troponin T, which has an essential role in Ca^2+^ signaling in the heart. Wang et al. conducted a GWAS in childhood cancer survivors with and without cardiomyopathy and identified an association of CELF4 SNP rs1786814 with susceptibility to ACT. In patients with an A allele (GA and AA genotypes), cardiomyopathy was infrequent and not dose related. However, among those patients exposed to > 300 mg/m^2^ of doxorubicin or equivalent, the rs1786814 GG genotype conferred a 10.2-fold increased risk of cardiomyopathy (95% CI: 3.8 to 27.3; *P* < 0.001) (13).

### Impaired Iron Metabolism

#### Hemochromatosis

Hemochromatosis (HFE) encodes a major histocompatibility complex MHC class 1–like protein that binds to transferrin. This protein regulates the production of hepcidin, a key regulator of the entry of iron into the circulation. Noting that dexrazoxane, an iron chelator, had a cardioprotective effect on patients receiving doxorubicin, Miranda et al. hypothesized that the gene responsible for human HFE could play a role in susceptibility to ACT. Indeed, HFE-deficient mice exhibited greater sensitivity to doxorubicin, with increased serum CK and mortality following chronic doxorubicin treatment (40). In a pediatric cohort where 10% carried the HFE SNP rs1800562 (p.C282Y) and the heterozygous rs1800562 (p.C282Y) genotype, the presence of these variants correlated with an increase in Troponin T and a reduction in fractional shortening at 2-year follow-up (41).

## Gene–Environment Interaction

The interaction between environmental factors (such as the anthracycline dose) and underlying genetic variation may affect the phenotypic expression of ACT. To date, most studies have not been designed to address this question in detail.

## Genetic Overlap Between Anthracycline-Associated Cardiotoxicity and Other Cardiomyocytes

**FIGURE 1:**
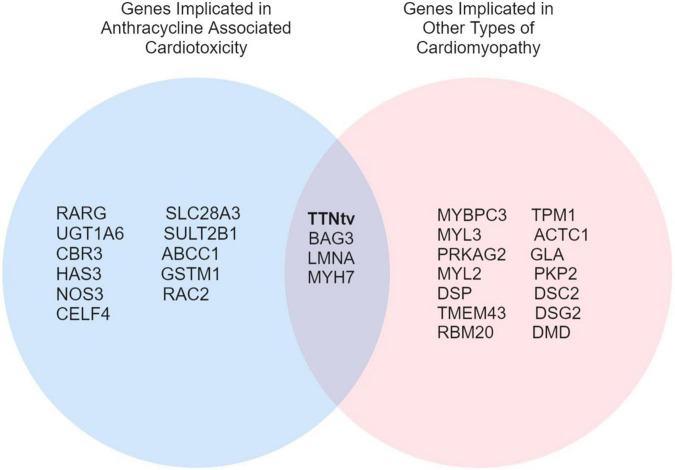
A Venn diagram of overlapping genes implicated in ACT and other types of cardiomyopathies.

Overlap in genetic variants between DCM and ACT represents a particularly interesting avenue of investigation (Figure 1). Garcia-Pavia et al. (14) demonstrated an increased prevalence of DCM-associated gene variants in the ACT patient population, where 12.2% of patients with ACT were found to have a cardiomyopathy variant. Titin-truncating variants (TTNtv) were identified in 16 of 213 ACT cases (7.5%) and were associated with more heart failure, hospitalizations, and atrial fibrillation, as occurs in patients with DCM caused by TTNtv (42, 43). Other genes associated with DCM such as BAG3, LMNA, and MYH7 have been poorly characterized in patients with ACT (14). As highlighted by the findings of Garcia-Pavia et al. (14), genetic modifiers of ACT overlapping with DCM genes may represent a distinct risk profile in addition to genes specific to ACT pathophysiology, a concept that will be important to consider as genetic testing is integrated into the clinical care of cardio-oncology patients.

## Type of Genetic Testing

Different types of genetic test are available to analyze changes in genes. At present, commercially available genetic tests do not include most of the genetic modifiers associated with ACT. Here we will focus on molecular testing that might be relevant to ACT gene testing in the future.

### Gene Panel Testing

Commercially available gene panels are able to identify specific genetic variants. The number and type of genes offered for sequencing are different between panels. The yield of a panel differs based on the number of genes within a panel, as was demonstrated by Pugh et al. (44). While evaluating 766 patients with DCM, a large gene panel of 47 genes identified an underlying culprit gene in 37% of patients, whereas the yield was less than 10% with a smaller gene panel (44). Broader gene panels have an advantage in evaluating a genetically and clinically heterogeneous disease, but this comes with the expense of a high rate of variants of uncertain significance (VUS). This has been challenged by recent data from Murphy et al. (45), where the most actionable variant in their cohort of patients with inherited cardiac conditions was detected in smaller gene panels. This suggests that larger gene panels may offer little extra sensitivity with a higher burden of VUS (45).

### Whole-Exome Sequencing

With the considerable decrease in cost, the clinical use of whole-exome sequencing has increased. Whole-exome sequencing can help identify novel variants that are not known to be involved in the pathogenesis of a disease (46). Importantly, whole-exome sequencing does not include most non-coding regions (intergenic areas and introns); however, these regions can be involved in the regulation of gene expression leading to disease pathogenesis (47). In the future, whole-genome sequencing may enable the characterization of coding and non-coding variation with minimal loss of sensitivity for pathogenic variants and increased opportunity to identify gene-gene or polygenic interactions that influence a patient’s risk of developing ACT, as has been done for other cardiovascular diseases (48, 49).

## Interpreting Genetic Test Reports

### American College of Medical Genetics and Genomics Guidelines

Differences in interpretation are present between laboratories as well as between clinicians. The American College of Medical Genetics and Genomics (ACMG) published a set of guidelines to provide consistent terminology. Using a four-tiered system, somatic sequence variations are categorized based on their clinical significance as being pathogenic, likely pathogenic, a VUS, likely benign or benign (50).

### Variants of Uncertain Significance: Determining Significance

Genetic testing is frequently inconclusive. This could be attributed to the absence of a pathological variant or the presence of a benign variant. Determining the pathogenesis of a variant using the ACMG criteria is important. At present, it is advised to treat variants in a binary fashion. Pathogenic variants and likely pathogenic variants are regarded as positive results. Benign variants and likely benign variants are considered to be negative results. VUSs that have not definitively been classified as pathogenic or benign are open to reclassification as new data become available. VUSs represent a challenge in genetic test interpretation, and this is especially true for ACT where most findings remain investigational. This further demonstrates the need for additional studies with larger sample size. Human induced pluripotent stem cell-derived cardiomyocytes (hiPSC-CMs) may be particularly useful in functional characterization of VUS (51).

## Genetic Testing Results and Potential Applications to Patient Management

There is no consensus on the clinical application of genetic testing in the management of patients receiving anthracyclines. With growing data to support genetic predisposition as a key factor in ACT, one might envision future risk assessment tools based on both genetic testing and clinical risk factors. For example, patients with the RARG rs2229774 risk variant and the UGT1A6*4 rs17863783 risk variant may be considered at increased risk of ACT beyond what would be estimated from clinical risk factors alone. This could prompt closer cardiovascular follow-up and echocardiographic imaging during and post-treatment, as well as more aggressive risk factor management.

At present, genetic evaluation has no clinical application in ACT evaluation, unless there is a family history of cardiomyopathy or sudden cardiac death that warrants further investigation. As more data emerge regarding genetic risk factors for ACT, clinicians could consider personalized approaches to cardioprotection as follows:

### Dexrazoxane

Dexrazoxane is an iron chelator that protects against oxidative stress. Several RCTs demonstrated the dexrazoxane is effective in preventing anthracycline cardiomyopathy and heart failure, although concern persists among many oncologists regarding mitigation of anthracycline antitumor efficacy and a possible increased risk of secondary malignancies, which has led to restriction of FDA approval for this agent.

### Liposomal Encapsulated Anthracyclines

The liposomal formulation of anthracyclines is thought to result in decreased drug delivery to cardiomyocytes, leading to decreased cardiotoxicity. Liposomal doxorubicin was found to have similar efficacy and survival outcomes as regular doxorubicin but with lower risk of ACT and congestive heart failure (52). However, the number of studies is small, and data on long-term follow-up are lacking.

### Primary Cardioprotective Agents

Several small RCTs have assessed the efficacy of neurohormonal blockade (beta blockers, ACEI, and ARBs) in preventing ACT (53). Although most suggest a modest attenuation of LVEF decline, these studies have largely been underpowered to detect differences in clinical heart failure. While routine use of neurohormonal blockade for primary cardioprotection is not supported by the current data, there may be some benefit in the setting of high-risk genetic variants and clinical features.

### Alternative Chemotherapy Regimens

In a patient with genetic variants that are expected to be high-risk for ACT, particularly if high doses of anthracyclines are anticipated, it may be reasonable to consider an alternative, less cardiotoxic chemotherapy regimen.

## Potential Uses of Genetic Testing in Patients Who Develop Anthracycline Associated Cardiotoxicity

In patients with established ACT, treatment is similar to that recommended by standard heart failure management guidelines. The use of neurohormonal blockade as the cornerstone of management is consistent with data demonstrating cardiac reverse remodeling and improved survival in heart failure in general. There has been interest in identifying which patients respond favorably to neurohormonal blockade. Outside the context of ACT, differences in individual response to beta-blockers are thought to be due to genetic polymorphisms in the gene encoding the beta-adrenergic receptor. In the WOSCOPS trial and MERIT-HF trial (54), these polymorphisms were not associated with a change in morbidity or mortality. Similarly, genes encoding angiotensin II and the associated receptors demonstrated polymorphisms associated with different response to therapy. For instance, a homozygous DD genotype for the ACE gene with a polymorphism of AGTR1 might cause higher levels of renin–angiotensin–aldosterone system activation, resulting in worse prognosis despite treatment with ACEI (55) and lower survival (56). The routine use of genetic testing for gene polymorphisms lacks clinical validation at present, but individualized medical therapy holds promise to improve outcomes in patient with ACT.

## Limitations of the Available Data

Most genetic studies of ACT have attempted to identify candidate genes and/or genetic modifiers by evaluating the cumulative burden of gene variants to determine which genes were overrepresented for genetic variation in a cohort of patients with ACT. This approach differs from that used for other inherited cardiac diseases in clinical practice, such as DCM, where the evidence for pathogenicity includes the evaluation of individuals SNPs based on prior reported associations for the SNP or location of SNPs in gene mutation “hotspots”. To achieve this level of evidence for individual SNPs in ACT will require utilization of genome editing tools such as CRISPR as well as patient-derived *in vitro* models such as hiPSC-CM, as well as accumulating evidence for genetic variants in ACT in clinical practice.

Furthermore, in other types of cardiomyopathy, the evaluation of variant pathogenicity is directed toward a monogenic variant that is otherwise absent (or of very low frequency) in the general population. However, reported variants associated with ACT have typically been common in the general population, with an allele frequency reaching up to 6% (e.g., ABCC2). These genetic modifiers may contribute to the risk of cardiomyopathy, but they likely have a low penetrance for disease in the general population and may require an environmental interaction (such as exposure to anthracycline) as a second hit to produce the clinical ACT phenotype. The uncertainty surrounding genetic testing will necessitate close collaboration with genetic counselors who can help navigate the implications of testing results as genetics is integrated into the cardio-oncology clinic.

## Future Directions

There continue to be several limitations to the use of genetics in routine clinical care. Above all, the identification of disease causing variants remains challenging. Some genetic variants might be necessary but not sufficient to predispose to ACT, and how these variants interact with other unidentified genetic factors remains to be explored. More studies are needed to identify high risk genetic variants with clinical validation. Recent data suggest that patient derived hiPSC-CM may provide a platform for validation of genes/variants identified through GWAS and to clarify the associated molecular mechanisms predisposing to cardiotoxicity. Ultimately, this approach may allow for tailored doses of chemotherapeutics based on patient genotype (57).

There has also been an increasing interest in understanding the role of complementary approaches, such as epigenetics, proteomics, and metabolomics in the development of cardiomyopathy. An integrated-omics approach has the potential to improve our understanding of ACT. Artificial intelligence methods could be applied to large populations to further improve the diagnosis, prognosis, and treatment of ACT. With improved understanding of genetic predisposition for ACT, a risk assessment model that incorporates genetics and conventional clinical risk factors may be better suited to classify an individual patient’s risk.

## Conclusion

Anthracycline-induced cardiomyopathy is a complex adverse drug reaction that is associated with morbidity, mortality, and increased social and economic burden for patients, their families, and the healthcare system. Recent advances in the field of genetics have led to an improved understanding of ACT, although significant limitations to clinical applicability remain.

## Author Contributions

AA: conceptualization. TA-O: analysis and data collection. TA-O and BW: writing original manuscript draft. AA and UT: review and editing manuscript. All authors reviewed the results and approved the final version of the manuscript.

## Conflict of Interest

AA has consulted for Sanofi and AstraZeneca, serves as an advisory board member for Cytokinetics, and serves as the principal investigator for a sponsored research agreement with Genentech. The remaining authors declare that the research was conducted in the absence of any commercial or financial relationships that could be construed as a potential conflict of interest.

## Publisher’s Note

All claims expressed in this article are solely those of the authors and do not necessarily represent those of their affiliated organizations, or those of the publisher, the editors and the reviewers. Any product that may be evaluated in this article, or claim that may be made by its manufacturer, is not guaranteed or endorsed by the publisher.
